# Underwater Electromagnetic Sensor Networks—Part I: Link Characterization †

**DOI:** 10.3390/s17010189

**Published:** 2017-01-19

**Authors:** Gara Quintana-Díaz, Pablo Mena-Rodríguez, Iván Pérez-Álvarez, Eugenio Jiménez, Blas-Pablo Dorta-Naranjo, Santiago Zazo, Marina Pérez, Eduardo Quevedo, Laura Cardona, J. Joaquín Hernández

**Affiliations:** 1 Instituto para el Desarrollo Tecnológico y la Innovación en Comunicaciones (IDeTIC), Universidad de Las Palmas de Gran Canaria (ULPGC), 35017 Las Palmas, Spain; pmena@idetic.eu (P.M.-R.); ivan.perez@ulpgc.es (I.P.-Á.); eugenio.jimenez@ulpgc.es (E.J.); pablo.dortanaranjo@ulpgc.es (B.-P.D.-N.); 2 Escuela Técnica Superior de Ingenieros de Telecomunicación (ETSIT) – C-303, Universidad Politécnica de Madrid (UPM), Av. Complutense 30, 28040 Madrid, Spain; santiago@gaps.ssr.upm.es (S.Z.); marina.perez@isom.upm.es (M.P.); 3 Plataforma Oceánica de Canarias (PLOCAN), 35214 Telde, Spain; eduardo.quevedo@plocan.eu (E.Q.); laura.cardona@plocan.eu (L.C.); joaquin.brito@plocan.eu (J.J.H.)

**Keywords:** underwater wireless sensor networks (UWSNs), electromagnetic, antennas, sea water, measurements, simulations, testbed

## Abstract

Underwater Wireless Sensor Networks (UWSNs) using electromagnetic (EM) technology in marine shallow waters are examined, not just for environmental monitoring but for further interesting applications. Particularly, the use of EM waves is reconsidered in shallow waters due to the benefits offered in this context, where acoustic and optical technologies have serious disadvantages. Sea water scenario is a harsh environment for radiocommunications, and there is no standard model for the underwater EM channel. The high conductivity of sea water, the effect of seabed and the surface make the behaviour of the channel hard to predict. This justifies the need of link characterization as the first step to approach the development of EM underwater sensor networks. To obtain a reliable link model, measurements and simulations are required. The measuring setup for this purpose is explained and described, as well as the procedures used. Several antennas have been designed and tested in low frequency bands. Agreement between attenuation measurements and simulations at different distances was analysed and made possible the validation of simulation setups and the design of different communications layers of the system. This leads to the second step of this work, where data and routing protocols for the sensor network are examined.

## 1. Introduction

Reliable underwater communications systems are required for many applications like tactical surveillance, pollution and environmental monitoring, oceanography research, among others. The latter ones are all related to the study of climate change, oceans and ecosystems. Valuable monitoring techniques would be extremely helpful to analyse constant variation of marine characteristics and human impact on these ecosystems. For example, the process of marine defaunation [1], which climate change is accelerating, requires efficient underwater monitoring. This is where underwater wireless sensor networks play an important role, as a solution to this problem, making possible communication between sensing nodes and periodic data retrieval.

However, the underwater scenario is a really harsh environment and different wireless technologies have to be examined. Acoustic communications are a standard for marine underwater communications and optical communications are proving to be useful in some underwater scenarios. Electromagnetic propagation has been studied for decades but just a few technologies have made it through. Acoustic systems are widely used for long distances in deep waters, but they have some drawbacks, especially in shallow waters, due to time-varying multipath propagation, Doppler shifting and Doppler spread [2]. Moreover, its bandwidth is limited to 5–7 KHz providing a low data rate. By contrast, optical technology can reach huge data rates (>1 Gbps) but is limited to short-range communications and clear waters [3]. Therefore, EM solutions are being re-evaluated for their use in shallow waters, where it can be useful to deploy a sensor network. Although EM waves suffer high attenuation in sea water, a series of advantages are offered, for instance, considerable bandwidth, fast propagation, immunity to turbidity, no multipath or Doppler propagation problems, and inexpensive and proven EM circuitry and antennas. It is widely accepted that the underwater acoustic channel is one of the most difficult types of communication media in use nowadays and high power systems have a negative impact on marine fauna [4]. In addition, optical technology is limited by water turbidity and ambient light noise, especially in coastal areas. Consequently, EM propagation has an increasing interest and is being studied by several research groups all over the world, hoping to establish it as a real alternative [5,6,7].

It is important to remark that different approaches in underwater radiocommunications systems lead to a wide range of results in literature. When analysing the state-of-the-art, there are five important aspects to highlight: antennas used, frequency bands, power transmitted, distances achieved and transmitter–receiver depths. In some cases, real condition experiments have been carried out. In others, simulations or theoretical approaches have been conducted, and, in a few cases, a combination of both is encountered. Regarding antennas, loop antennas and different types of dipoles are usually tested by research groups, but no agreement has been found on which is the best choice. Dipoles with and without insulated sections are examined in [7,8,9], whilst others carry out experiments with loop antennas [7]. Nevertheless, magnetic loop antennas seem to have a certain acceptance. According to Somaraju and Trumpf [10], propagation over long distances at frequencies of MHz is feasible because water conductivity decreases at certain frequencies, yet this has not be proven experimentally. Accordingly, Shaw and Al-Shamma [7] showed the possibility of communicating at 1 MHz over 90 m in a real sea water experiment, but no additional measurements have been published to validate these results in sea water. Instead, their research line was changed to fresh water in the last papers. Dipoles and loop antennas were tested. However, the latter seemed to be more successful. On the contrary, in [8], it is stated the need of using low frequency carriers, up to 20 KHz, due to the dramatic rise in attenuation as frequency increases. In this case, with vertical dipoles, distances of up to 20 m were reached in real environment experiments, and these results were compared to theoretical ones. These antennas were also employed in [9], at 100 KHz and 14 MHz, in order to validate theoretical solutions with experimental measurements in near and intermediate field conditions. According to Lucas and Yip [11], a parallel dipole antenna contained within a water-filled PVC barrel could excite water molecules to emit dipole radiation in seawater. All of these works can give the reader a view of the variety of the approaches in this field and how difficult it is to design an experiment based on their different opinions.

Electromagnetic underwater communications are definitely not a mature technology and require further research to start the development of a reliable body of knowledge. Different researchers try different approaches. Therefore, there is an urgent need to characterize the underwater channel before attempting the development of underwater sensor networks. A productive way of doing so is to carry out measurements and simulations. Measurements would validate simulation tools and this would make possible conducting additional simulations reducing the need of going to field sites. Reliable simulation tools are extremely useful as several scenarios can be easily tested.

Due to this lack of consolidation of underwater communication in the scientific field and the great potential of radiofrequency communication in shallow waters, in 2014, a project entitled UNDERWater radiocommunications for Optimized monitoring using multiReLay Devices (UNDERWORLD) was proposed. Its aim was and is to re-evaluate the possibility of using EM communications in underwater wireless sensor networks, and the first results can be found in [12,13]. Nodes for this network should require low power to extend battery life, should not be too bulky and predicted distance between nodes is around 15–30 m. This network will be useful for monitoring shallow waters from the seabed. For instance, where air RF communications are not practical. The rest of the paper is organized as follows.

Firstly, experimental measurements are explained in detail, starting with the description of the measurement site. Experimental testbed, which is divided into two parts, is extremely important in order to achieve reliable results and that is why it is fully described. This includes the measuring system, as well as monitoring and control of the testbed. Another important aspect of the experiments, as it has been discussed previously, is the set of antennas employed and which one shows better performance. Afterwards, setup procedures will be explained, not just for launching equipments, but for measuring.

Secondly, a great variety of results is presented. The most remarkable aspect of this work is that real marine measurements were performed, with several kinds of antennas and a reliable testbed, and, at the same time, simulations were carried out in the laboratory, achieving high agreement between both methods. Thus, simulation setup parameters and the design of the testbed were also verified. Even though sea water EM properties have been known for decades and several practical and theoretical works have been published, there is no consensus in literature on how to characterize the underwater channel. Therefore, hard work is still needed. A comparison between two types of underwater models for simulations is made in order to decide which one is more appropriate. The performance of dipole and loop antennas is discussed with the support of simulations and real measurements. A characterization of the underwater link is achieved with the latter antennas for frequencies ranging from 10 KHz to 1 MHz, since agreement between simulations and measurements has been obtained. This agreement enables the validation of simulation tools and setup parameters. Frequencies of minimum attenuation and its corresponding values of attenuation are simulated for distances up to 30 m.

Finally, relevant conclusions have been drawn and discrepancies with other authors are presented. Future research lines are also introduced.

## 2. Experimental Section

### 2.1. Measurement Site

**Figure 1:**
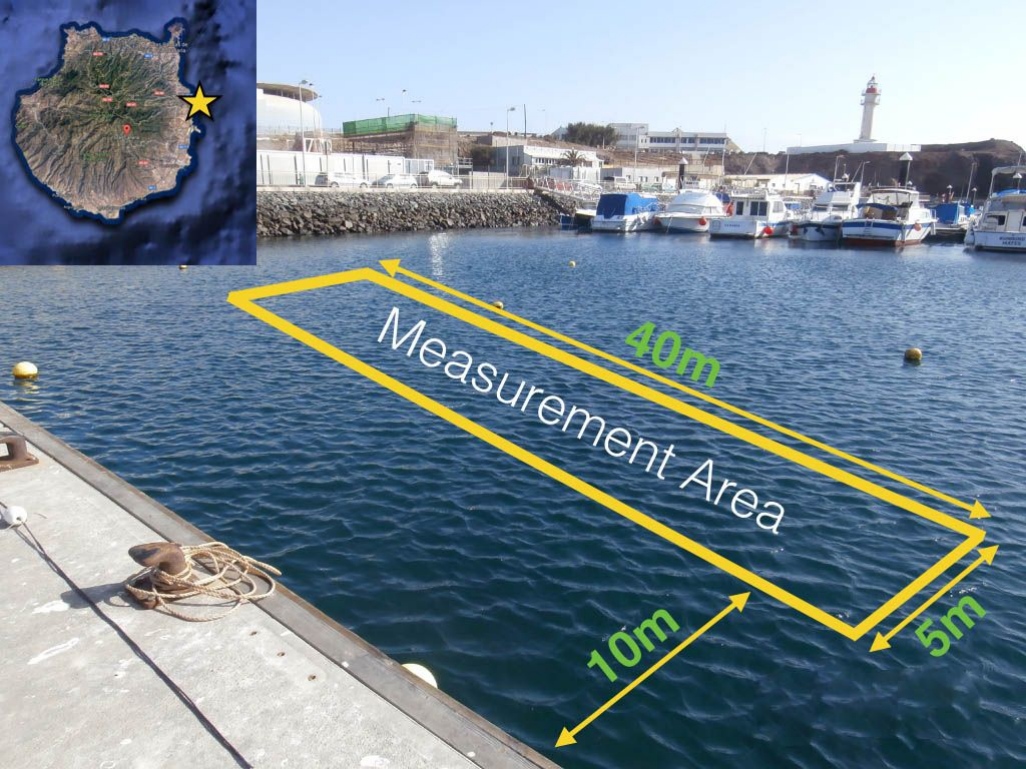
Measurement area in Taliarte’s harbour.

These campaigns aimed the characterization of the underwater EM link at carrying out measurements in real conditions. Thus, experiments were performed in Taliarte’s harbour near PLOCAN’s facilities in Gran Canaria (Spain). Measurement area had dimensions of 40 m × 5 m and was separated 15 m from a concrete floating pier that was in line with it (Figure 1). Monitoring and control of the instruments were performed on this pier.

The measurement site is surrounded by boats and other typical harbour objects. Moreover, there are concrete blocks lying on the seabed. The effect of these obstacles was found negligible for the measurements and only seabed composition and water depth have been studied and introduced as parameters in simulation tools. Water depth varied from 4 to 5.5 m depending on tides, and there was a sandy seabed.

### 2.2. Testbed

#### 2.2.1. Measuring System

**Figure 2:**
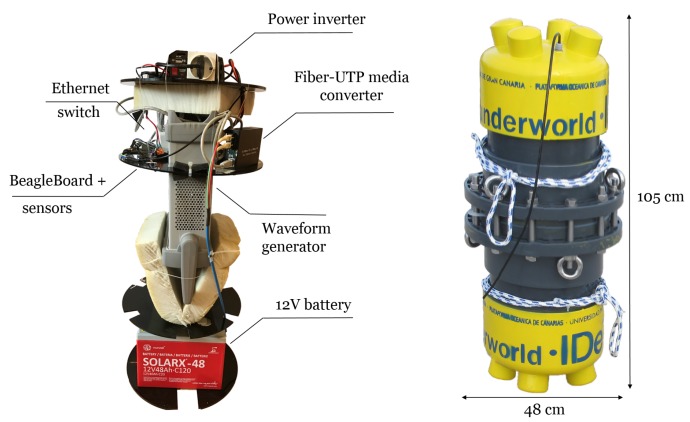
Transmitter chamber (the inside on the **left** side and the outside on the **right** side).

**Figure 3:**
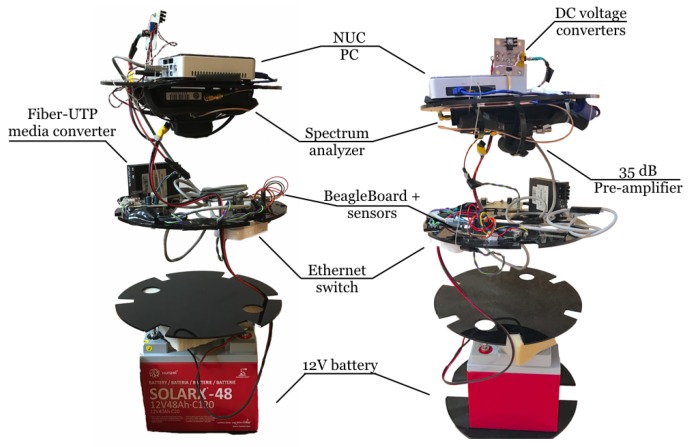
Receiver chamber (front and lateral view of the inside).

It is important to notice that our testbed was designed to take advantage of reliable and well-known laboratory instruments, instead of customized devices, and the whole system was meant to adapt these instruments to work underwater. Hence, the measuring system consists of two sealed chambers built with large PVC pipes that contain several instruments: one is the transmitter and the other one is the receiver. Both chambers include a coaxial cable to connect antennas to the instruments inside. Figure 2 and Figure 3 show the interior structure, circuitry and instruments used in the transmitter and receiver, respectively.

A bottom-to-top description of the transmitter chamber is now detailed and images of the interior and exterior are labelled in Figure 2. A 12 V battery is placed at the bottom between two methacrylate disk-shaped sheets. The next section holds a waveform generator (Keysight 33220A, Keysight Technologies Inc., Santa Rosa, CA, USA) controlled by Ethernet, which is needed to transmit signals. All instruments and battery are surrounded by foam rubber to avoid movement inside the container. On the disk above, temperature and air pressure sensors are monitored by a BeagleBone Black board running a Linux distribution and connected to the Ethernet switch. This device is also connected to the Fiber-UTP media converter, so that instruments can be controlled through a long fiber link with no coupling problems between transmitter and receiver cables. On top, there is a power inverter that provides AC current to the signal generator.

Both chambers are formed by a PVC pipe of 30 cm interior diameter ended with two caps and a dresser coupling in the middle. Each chamber weighs approximately 80 kg when full and the exterior measures of the cylinder are 105 cm × 48 cm.

Receiver instruments in Figure 3 (front and lateral view) have an analogous layout. A similar battery is also located at the bottom. The section above was designed to hold a hand-held spectrum analyser (Agilent N9340B, Agilent Technologies Co Ltd., Santa Clara, CA, USA), but in current measurements this model is not used anymore due to its minimum frequency limitations and sensitivity. This layer has the same elements as in that of the transmitter. The subsequent disk contains the currently used spectrum analyser, the Aaronia GmbH (Gewerbegebiet Aaronia AG, Strickscheid, Germany) (1 Hz to 30 MHz), which comprises a 35 dB pre-amplifier and an Intel NUC (Intel Co., Santa Clara, CA, USA) PC. The Aaronia spectrum analyser requires a USB connection to a PC. For this reason, an Intel NUC was mounted inside the chamber running Windows 7 (Microsoft Co., Redmond, WA, USA) and Aaronia software. Finally, in order to supply different voltage requirements for the devices (5 and 12 V) DC voltage converters were implemented in a board with a heat-sink.

The last version of the receptacles includes four cables that pass through the pipe by cable glands and have their own plastic covering. When the fitting was screwed down, sealing grommets around the cable provided a watertight seal. These four cables provide:

- Access to the battery for charging;
- ON/OFF switch;
- Fiber cable link to control equipment from the pier;
- Coaxial for antenna connection.

The first two are not used while submerged, thus water sealed terminations were built with screwed small pipes and tapped unions. In addition, chambers were pressurized to provide appropriate water sealing and avoid water getting inside. Therefore, air valves were built on the sides of both capsules.

#### 2.2.2. Monitoring and Control

Monitoring and control of the testbed include: checking pressure and temperature values provided by interior sensors, modifying power transmitted, changing frequency sweeps and retrieving data from the spectrum analyzer.

Instruments inside the chambers are controlled thanks to two optic fiber links. Each fiber is connected on one side to a Fiber-UTP media converter that lies on a table on the pier, and on the other side to another converter with the same characteristics inside each chamber (Figure 3). Both are then connected to a switch which creates a Local Area Network (LAN) and includes instruments from both chambers and the controlling laptop. Fiber link with both submarine capsules assured no signal leaking or coupling from or to the instruments.

### 2.3. Set of Antennas

**Figure 4:**
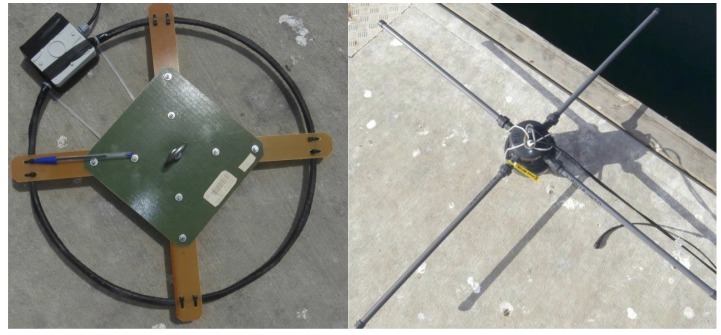
Antenna examples: on the **left** 22 cm magnetic loop and insulated crossed-dipole on the **right**.

There is no standard antenna type to use in each situation for underwater radiocommunications. This is why in this work various antennas have been tested, in order to find out which one is appropriate for our sensor network. Basically, we tested electric dipoles and magnetic dipoles and four configurations (Figure 4):

- Insulated dipoles;
- Insulated dipoles with uninsulated tips;
- Magnetic loops of 22 and 40 cm.

Dipole antennas have been designed as a body with two screwed elements surrounded by a tight PVC pipe screwed to the body as well. Each element was 59 cm long and was made to be easily unmounted to transport or be replaced with diverse kinds of terminations. This enables the possibility of using two types of dipoles: completely insulated bars [14] and insulated with uninsulated terminations [6]. The body of the dipole was built with high pressure pipe parts leaving a cavity inside for the connection with coaxials and a balun circuit. The reason for using dipole antennas, apart from being widely used in literature, is that they allow an easy deployment, and crossed dipoles could permit a comfortable orientation. A conduction phenomenon was found when using uninsulated dipoles in sea water, producing high losses. Therefore, these antennas were not used in harbour’s experiments [13]. In addition, loop antennas were built to be able to compare their performances and because some authors consider these as the most suitable antennas for underwater communications [7]. Two versions were designed and tested, a pair with a radius of 22 cm and another with 40 cm to assess the effect of size increase. Loop antennas were built with 10 turns of enamelled copper wire (coating insulation). All copper turns were packed together and covered with self-vulcanizing tape for protection, and a balun was used for a balanced excitation.

After several tests in the field, dipole antennas showed very poor performance (Section 3.2) compared to magnetic loops, in line with some literature [15]. Magnetic loop antennas were selected, and all the experimental results in this paper were obtained with them.

### 2.4. Setup Procedures

#### 2.4.1. Launching Procedure

**Figure 5:**
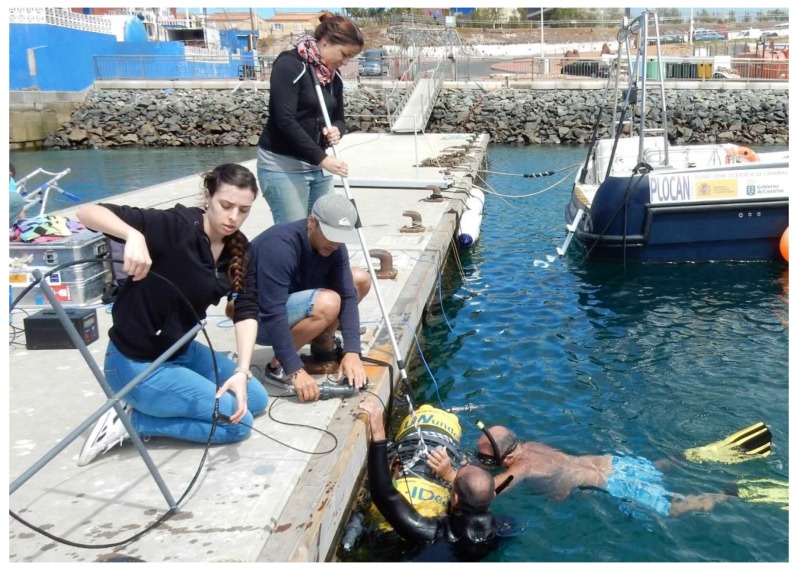
Launching procedure.

Before performing underwater experiments, some measurements are carried out on the pier in order to test air propagation between antennas and verify testbed behaviour. Underwater measurements start with the launching of each chamber with its antenna connected (Figure 5). For this purpose, a small marine crane is required to hold the containers and throw them into the sea, as each chamber weighs approximately 80 kg. Then, a diver locates the container and the antenna on the seabed at a specific separation from the other one with the help of a measuring rope.

Three types of antenna scenarios are of great interest in this work (the first two take place underwater):

- Variation of horizontal separation between the centres of antennas;
- Variation of vertical separation;
- Sea to air measurements.

These scenarios require different antenna movements. Nevertheless, only the horizontal separation measurements are finished and explained in this work. The others are currently in progress. Horizontal variation implies both transmitter and receiver on the seabed and the separation between them is increased or decreased by locating one of them farther. However, dipoles and loop antennas are always on the seabed. Dipoles are set to use horizontal polarization and are parallel to each other, while loop antennas are both lying on the seabed. Regarding the alignment, both loops have been placed on the seabed, ideally a horizontal plane in order to have nearly omnidirectional coverage. A measuring rope assisted the placement of the antennas. However, high precision is not achieved due to the unevenness of the seabed, especially at very short distances.

By contrast, vertical variation is planned to be performed when antennas are separated a fixed distance horizontally, and a reference measurement is carried out when both of them are on the seabed. Subsequent measurements will consist of lifting one of them metre by metre to analyse the behaviour of the channel under this condition (vertical separation). The last measurement will be conducted with one antenna still on the seabed and at the same horizontal distance while the other is suspended 0.5 m above the sea surface. Afterwards, they will swap places to check channel isotropy.

#### 2.4.2. Measuring Procedure

One of the most important tasks when attempting underwater channel characterization is conducting noise floor measurements, as they are strictly necessary to assess possible interferences and to discuss sensitivity issues. In this work, they were performed with each type of antenna prior to signal transmission. It was then that reliable measurements of attenuation could be carried out.

**Figure 6:**
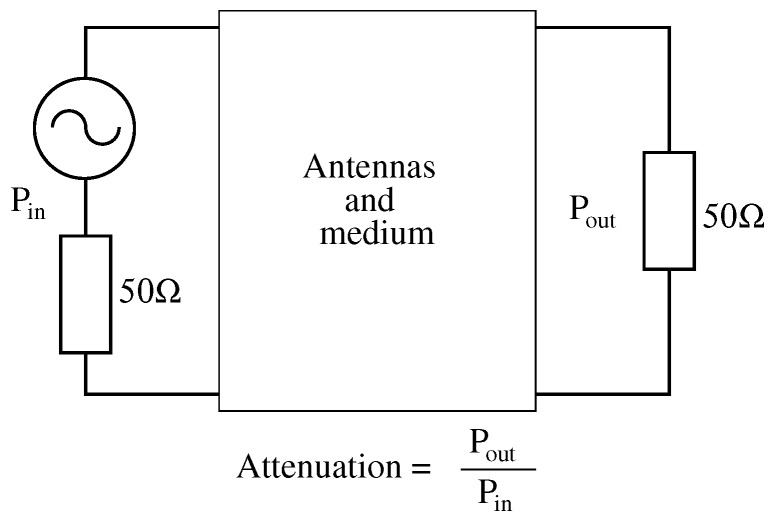
Attenuation description.

It is important to explain how attenuation is understood in this context, as power dissipated (P_out_) by a load divided by power transfer (P_in_) from the generator (Figure 6). Therefore, this attenuation involves antenna, cable and device losses. In short, it refers to all losses suffered by signals between the output of the signal generator and the input of the spectrum analyzer.

Depending on the type of antennas tested and the separation between them, different power ranges were used. Low power (0–10 dBm) was employed for short distances when testing loop antennas, in order to avoid saturation of electronic equipment, as strong signals were received in this case, whereas 23 dBm was the maximum power supplied by the signal generator, and it was just employed for dipoles as the last resource to get the maximum feasible distance. The usual power was 18 dBm to avoid harmonic distortion. Regarding transmitted frequencies, three bands were tested, especially the first two:

- 10–100 KHz;
- 100 KHz–1 MHz;
- 1–20 MHz.

During preliminary frequency measurements, frequency sweeps were performed up to 20 MHz to reach the maximum frequency that could be used. Nevertheless, no signal was received in such a high band under our measurements conditions. This is the reason why the upper limit for characterization was established in 1 MHz. To obtain higher resolution, the spectrum below 1 MHz was divided into two bands 10–100 KHz and 100 KHz–1 MHz.

In the frequency response measurement procedure, a sinusoidal signal was transmitted and constantly swept between a range of frequencies. At the same time, data was retrieved from the spectrum analyzer in raw recordings of all sweeps in a time-frequency matrix of power data. This enabled easy post-processing and graph generation. Looking for maximum received power in each frequency, we were able to obtain attenuation curves.

Time variation measurements were carried out with antennas in a fixed position, placed on the seabed and separated 4 m. A programme was employed to vary sine frequency to 40, 46 and 90 KHz each 10 s over a period of two hours.

### 2.5. Simulation Procedures

Measurements aimed at the validation of simulation tools and their setup parameters, which are carried out using a commercial MoM (Method of Moments) solver: FEKO. This solver supports the main characteristics required for this analysis: planar green functions for multilayered media, dielectric coated wires and special basis functions for low frequency analysis. Once measurements agree with simulation results, some measurements in the harbour can be avoided, as FEKO will simulate environmental and technical features precisely.

Carrying out simulations for RF underwater communications is solving an electromagnetic problem. Therefore, the first things to decide are frequency bands and distances of interest. Typically, simulations are carried out from 10 KHz to 1 MHz varying distance from 2 to 13 m in 1 m steps. Regarding simulations setup, the most simple case is when the medium is homogeneous, that is, sea water or free-space to start with. This is explained in previous papers [12] where its inaccuracy was proved. In order to get close to a real environment, a layered medium was also simulated: sea water with seabed. According to [6,16], sea water can be modelled as a dielectric with conductivity *σ* = 4.5 S/m and permittivity $\epsilon_{r}$ = 81. Seabed, fine sand, is modelled as a dielectric with permittivity $\epsilon_{r}$ = 3.5 (the effect of the value of permittivity on our simulations is negligible since it is a magnetoquasistatic problem) and conductivity *σ* = 1 S/m. The purpose of this last simulation was to determine the influence of the sea water–seabed interface in the wave propagation.

## 3. Results

### 3.1. Seabed Influence in Simulations

**Figure 7:**
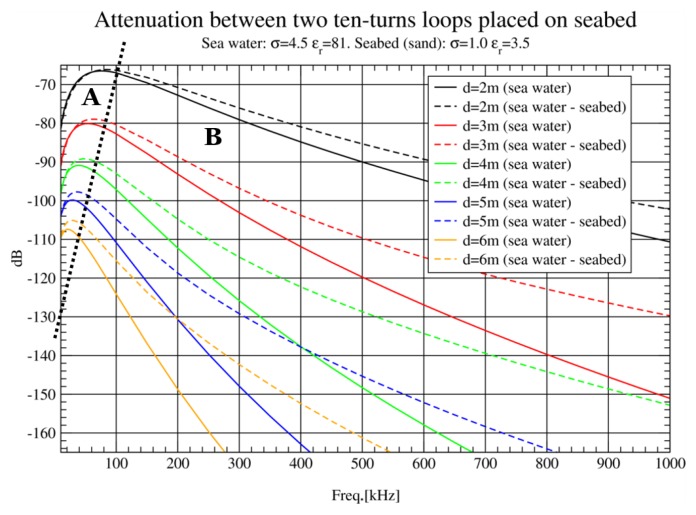
Influence of seabed layer inclusion in simulations.

The results from simulation of antennas in sea water and simulations of antennas placed on seabed at several distances (d) are compared in Figure 7. As it can be seen, sea water attenuation is dramatically increased as frequencies are higher, which is coherent with theoretical graphs in literature [8]. Moreover, there are clearly two distinguishable areas, A and B, separated by a black dashed line in the figure. Side A is defined by frequency bands where attenuation is not significantly influenced by seabed layer, whereas B comprises the spectrum area where attenuation is greatly affected by the seabed. It can be inferred that, in this case, energy is travelling through the sea-to-seabed interface, as the effect of the seabed layer is to decrease the attenuation at all frequencies. However, for low frequencies and distances, or for very low frequencies, attenuation seems to be independent from the medium as eddy currents are not very important.

Another interesting feature that can be extracted from this graph is the displacement of minimum attenuation with the distance. The longer the distance between nodes, the lower frequency for minimum attenuation values. This effect is studied in more detail in Section 3.5.

### 3.2. Electric Dipole Performance

Several measurements were made with insulated dipoles described in Section 2.3. However, the performance of these antennas was very disappointing. Dipole received power was tenths of decibels below loop antennas in all distances and scenarios tested. The signal generator had to be forced to transmit its maximum amplitude (23 dBm of power) when the antennas were separated just 1 m. Therefore, they were dismissed for further experiments.

In addition, some experiments were carried out using insulated dipoles with uninsulated terminations, that is, with 1 cm of both ends of the dipole elements in direct contact with sea water. In this case, distances of 6 m were reached before signal disappeared below our instrument noise floor (≈−120 dBm), so it did not fulfill the network distance requirements. Moreover, loop antenna attenuation was up to 20 dB lower at 1 m, for instance. Having said that, these dipole antennas were also dismissed.

### 3.3. Simulated Impact of Loop Antenna Radius

In this section, the influence of increasing loop antenna radius is assessed. Theoretically, as antennas cover a larger area, more magnetic flux is captured and more voltage is induced to antennas. Therefore, by designing loop antennas with 22 and 40 cm radius, almost double the size, performance should improve considerably.

**Figure 8:**
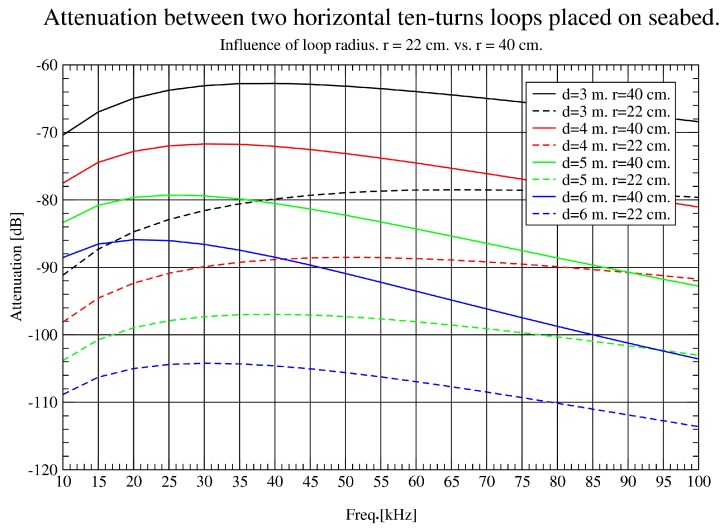
Influence of loop radius (10 KHz–100 KHz).

**Figure 9:**
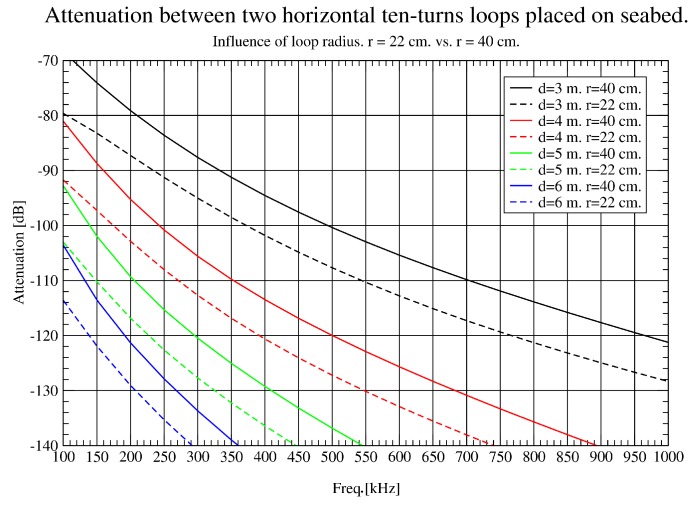
Influence of loop radius (100 KHz–1 MHz).

In Figure 8, the effect of doubling the loop radius in the lower band studied (10–100 KHz) is shown. An attenuation reduction of about 15 dB at 3 m is obtained and even more improvement is achieved at longer distances. This nonlinear behaviour could be explained due to the effect of the seabed. For the upper band (Figure 9), there is also an improvement, but not as dramatic as for the lower band, observing a constant gain of 7 dB approximately.

To sum up, bigger loop radius enables communications over longer distances and 40 cm was found to be a good compromise between distance requirements and network node size. As attenuation in 10–100 KHz band is lower in all experiments performed, it was chosen as the band of interest in this project.

### 3.4. Validation of Simulations with Horizontal Variations

**Figure 10:**
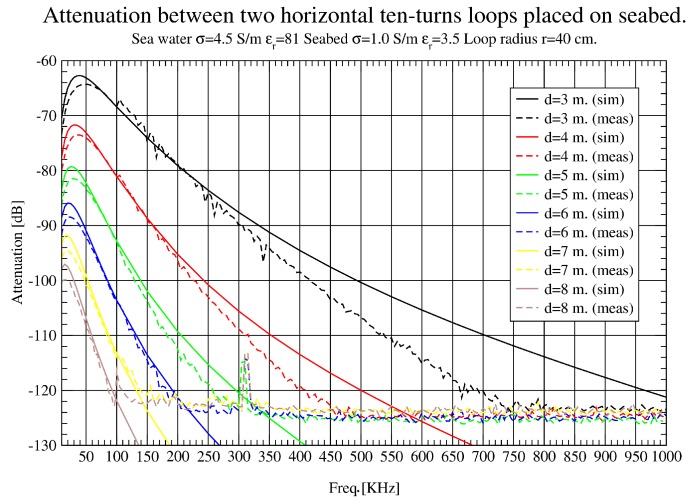
Path loss (dB) comparison up to 8 m (10 KHz–1 MHz).

**Figure 11:**
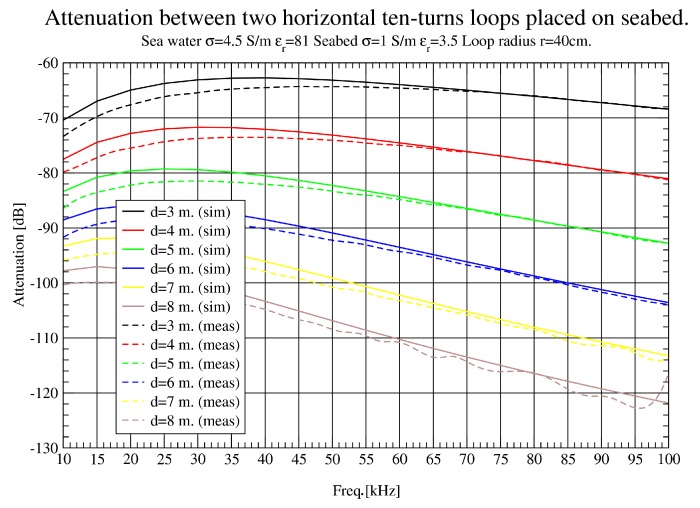
Path loss (dB) comparison up to 8 m (10 KHz–100 KHz).

**Figure 12:**
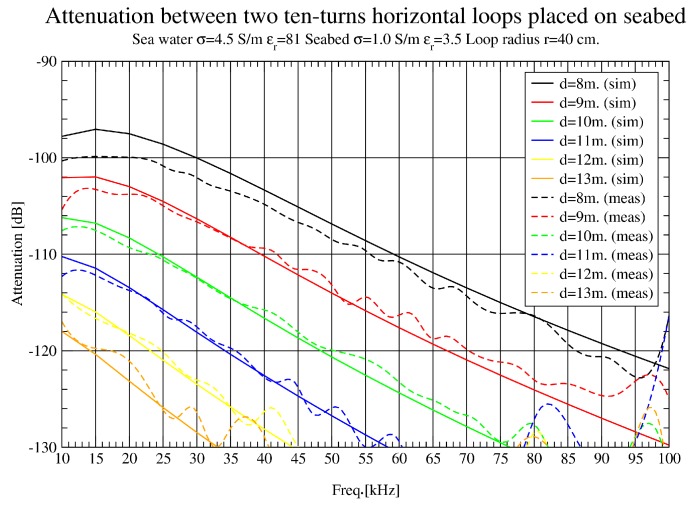
Path loss (dB) comparison up to 13 m (10 KHz–100 KHz).

In Figure 10, Figure 11 and Figure 12, attenuation measured with loop antennas of 40 cm radius is shown, as well as simulation results for horizontal antenna movements. In Figure 10, a full sweep from 10 KHz to 1 MHz is presented. Very good agreement between simulation and measurement results is obtained, except for the areas of higher frequencies and short distances. In this case, the differences are caused by two factors: the struggle of locating antennas precisely at short distances where the errors are proportionally higher and the tremendous attenuation suffered in higher frequencies. As attenuation is greater in this band, a small error of position leads to enormous variation in attenuation values. It is important to notice that the instruments’ noise floor is around −125 dBm, measured with an RBW (Resolution Bandwidth) of 3 KHz and a 50 Ω load. Loop antenna in air captures significantly more noise level while, submerged on the seabed, the noise level is around −125 dBm again. It was found that the dominant noise is generated within the measuring equipment, and most ambient noise captured by the antenna in the seabed is below that limit. This noise floor limits the smallest signal power that can be measured. It was flat and maintained the same value during several measuring campaigns.

In Figure 11, a zoom in the lower band is presented to analyse the results at 3–8 m. There is a remarkable agreement between simulation and measurement results, which makes possible the validation of simulation tools. Even in Figure 12 (8–13 m), some interference appears around 90 KHz.

### 3.5. Optimum Frequency for a Given Distance

**Figure 13:**
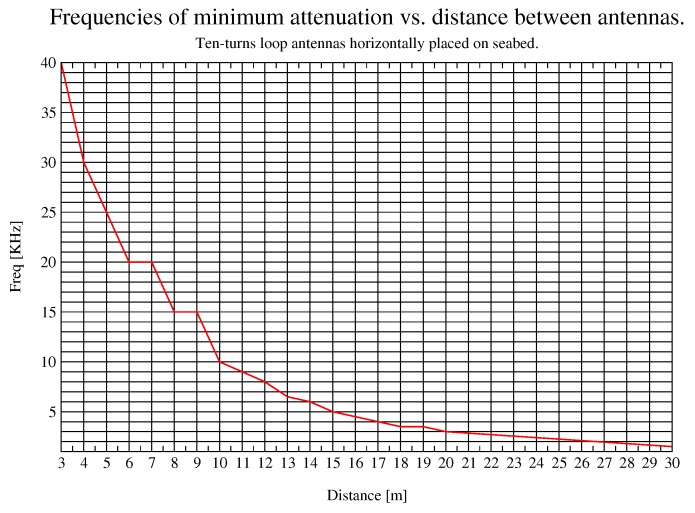
Optimum frequency for a given distance.

In this work, the frequency with less attenuation is referred to as the optimum frequency. This frequency is valuable as carrier frequency for a system that must optimize energy consumption. Figure 10 shows that there is a frequency with minimum attenuation for each distance. In other words, there is a local minimum for each distance, which means that lower and higher frequencies than this one will present higher attenuation at that distance. This optimum frequency value reduces as the distance between antennas increases, that is, it is shifted towards lower frequencies. This effect is also presented in simulation results for further distances (Figure 13), where optimum frequencies start with a value of nearly 40 KHz at 3 m and reaches 2 KHz at 26 m. These results lead to a compromise when designing the communication system of the sensor network, either more bandwidth or longer distances between nodes.

**Figure 14:**
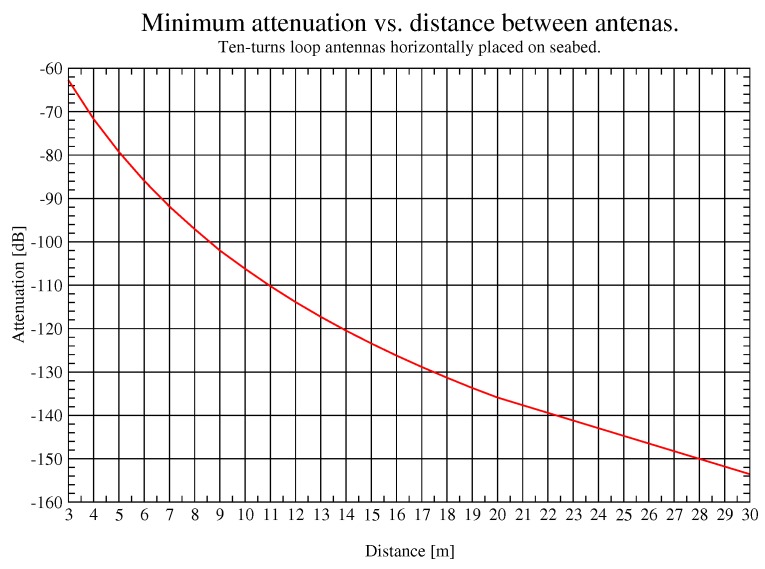
Optimum frequency for a given distance.

In addition, in Figure 14, minimum attenuation for the previously mentioned frequencies is shown. This value ranges from 67 dB at 3 m to 154 dB at 30 m, but it is important to remember that in this work attenuation includes all losses of the testbed and path loss of the link. Taking into account these results, if nodes transmit 35 dBm of power and their sensitivity is around −120 dBm, achieving communications over 30 m is feasible varying the optimum frequency.

### 3.6. Channel Time Variation

**Figure 15:**
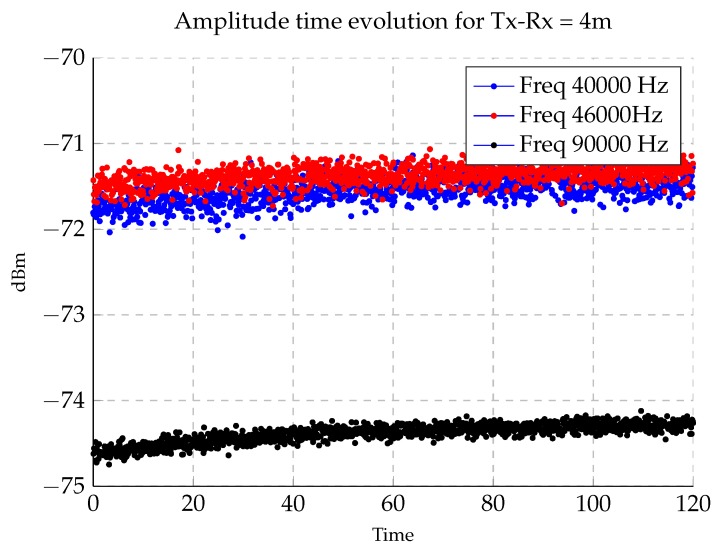
Amplitude time evolution during a 2 h time period.

Channel characterization requires not just frequency domain analysis but also time domain in order to check Doppler spread. As explained in the experimental section, time variation of the underwater channel was assessed. In Figure 15, it can be seen that no major variation took place during a 2 h time period. A small shift of about 0.5 dB was detected in this period. After analysing this small shift, temperature stabilization inside the chamber was concluded to be the cause. Therefore, the underwater channel is proved to be time-invariant, agreeing with [2].

## 4. Discussion and Conclusions

In this paper, a full description of a testbed for underwater communications in a real environment is provided. Moreover, a characterization of the underwater link in the frequency domain has been obtained after carrying out several measurements in underwater scenarios with dipole and loop antennas, obtaining enough information to decide some basic parameters of the communication system. Since good agreement between measurements and simulation results has been achieved, simulation tools and their setup parameters have been validated. Therefore, further experiments can be simulated.

Thanks to this study, several important results have been obtained, making possible the design of the physical layer of the UWSN. Loop antennas were found to be the most useful ones for shallow waters. Moreover, a 40 cm radius can be an adequate size, as there are considerably lower losses than with 22 cm, and it is not too large to be used in a UWSN. Frequency response figures between 10 KHz to 1 MHz for different distances have been provided, making these results a useful tool for further research on the field. Possible carrier frequencies for sensor nodes and maximum distances that can be reached have been simulated.

Regarding future research lines, the next step is to carry out experiments with ferrite antennas, which are being designed at the moment, and may offer the same performance with a smaller form factor. Furthermore, node prototypes for the UWSN are currently in development.

## Abbreviations

The following abbreviations are used in this manuscript:

UWSNs	Underwater Wireless Sensor Networks
EM	Electromagnetic
MoM	Method of Moments
PVC	Polyvinyl chloride
RF	Radio Frequency
UTP	Unshielded Twisted Pair
DC	Direct Current
FEKO	FEldberechnung für Körper mit beliebiger Oberfläche
RBW	Resolution Bandwidth
